# Yogurt Enriched with Omega-3 Fatty Acids

**DOI:** 10.3390/foods15091460

**Published:** 2026-04-22

**Authors:** Milena Savatinova, Mihaela Ivanova, Krastena Nikolova, Ivan Ivanov, Natalina Panova

**Affiliations:** 1 Department of Technology of Milk and Dairy Products, University of Food Technologies, 26 Maritsa Blvd., 4002 Plovdiv, Bulgaria; mivanova@uft-plovdiv.bg; 2 Department of Physics and Biophysics, Medical University-Varna, 55 Marin Drinov Str., 9000 Varna, Bulgaria; panova@mu-varna.bg; 3 Department of Organic Chemistry and Inorganic Chemistry, University of Food Technologies, 26 Maritsa Blvd., 4002 Plovdiv, Bulgaria; ivanov_ivan.1979@yahoo.com

**Keywords:** foods enriched with omega-3 fatty acids, dairy products, omega-3 enrichment, vegetable oils, marine oil, alginate beads, storage stability

## Abstract

Yogurt represents a traditional fermented dairy product characteristic of the Balkan Peninsula and is widely consumed in the Republic of Bulgaria. The aim of the present study was to develop omega-3-enriched yogurt. Four yogurts were produced: one control sample and three experimental variants enriched with chia oil (0.63%), cod liver oil (1.55%), and algal oil (1.10%). Coriander essential oil (0.038%) was added to each oil formulation. The products were monitored on days 1 and 14 of storage. The oils were pre-encapsulated in alginate beads to limit oxidative processes and preserve sensory properties. Yogurt samples were evaluated for oxidative stability, fatty acid composition, microbiological parameters, physicochemical properties, textural and sensory characteristics. Titratable acidity, pH, water-holding capacity, antioxidant activity, and microbiological parameters were not significantly affected by the incorporation of encapsulated oils. In contrast, significant differences were observed in texture and sensory attributes among the enriched variants. The chia oil sample exhibited the highest oxidative stability, followed by the algal oil yogurt, whereas the lowest stability was observed in the cod liver oil variant; however, all products remained within acceptable oxidation limits up to day 14. Approximately 350 g, 260 g, and 120 g of yogurt enriched with chia, cod liver, and algal oil, respectively, were required to meet the recommended daily omega-3 intake. The developed products demonstrated potential as dairy foods enriched with omega-3 fatty acids, with improved nutritional value.

## 1. Introduction

Foods enriched with bioactive compounds have been increasingly investigated due to their potential to provide health-related benefits beyond basic nutrition. Among these, yogurt is widely recognized as a suitable matrix for the incorporation of bioactive compounds owing to its favorable nutritional profile, probiotic content, and high digestibility. Yogurt is produced through lactic acid fermentation, during which *Lactobacillus* *delbrueckii* subsp. bulgaricus and *Streptococcus thermophilus* convert lactose into lactic acid and other metabolites, contributing to the characteristic texture and flavor of the product [1].

Despite its nutritional advantages, yogurt naturally contains low levels of omega-3 fatty acids, which are well known for their beneficial effects on cardiovascular and metabolic health. Omega-3 fatty acids, including α-linolenic acid (ALA), eicosapentaenoic acid (EPA), and docosahexaenoic acid (DHA), have been associated with anti-inflammatory effects and improved lipid metabolism [2]. Consequently, the enrichment of dairy products with omega-3 fatty acids has attracted increasing scientific and industrial interest [3]. However, the incorporation of omega-3 fatty acids into yogurt presents significant technological challenges due to their low water solubility, susceptibility to oxidation, and potential to generate undesirable off-flavors, which may negatively affect product stability and sensory quality [4]. Previous studies have demonstrated that the use of emulsified or encapsulated omega-3 oils can improve physicochemical properties and oxidative stability, although sensory optimization remains a critical factor for consumer acceptance [4,5]. Encapsulation techniques, particularly those based on biopolymers such as alginate, have been widely investigated as effective approaches to protect sensitive lipophilic compounds and to control their release within food systems. Alginate-based encapsulation has been shown to enhance oxidative stability and reduce unfavorable flavor interactions, thereby improving the overall quality of enriched dairy products [6]. In addition, both plant-derived oils (e.g., chia oil) and marine sources (e.g., fish oil and algal oil) are commonly used for omega-3 enrichment, each presenting specific compositional and technological characteristics [7,8]. Although considerable progress has been made in the development of omega-3-enriched dairy products, challenges remain in balancing nutritional enhancement with sensory acceptability, as well as in understanding the interactions between lipids, proteins, and polysaccharides within the dairy matrix. Furthermore, limited information is available regarding omega-3 enrichment of traditional Bulgarian yogurt, particularly using encapsulated systems.

Therefore, the present study aims to develop yogurt enriched with omega-3 fatty acids via alginate beads containing chia, algal, and cod liver oils, and to evaluate the physicochemical, oxidative, fatty acid, and sensory characteristics of the fortified yogurts in comparison with a control sample.

## 2. Materials and Methods

### 2.1. Milk

Fresh milk was delivered by a certified supplier of the University of Food

Technology, Plovdiv. The milk (10 mL sample) was analysed using an automatic milk analyzer MilkoScope Expert (Etcon Analytical, Lagos, Nigeria) at room temperature (20 ± 2 °C). The obtained physicochemical parameters of the milk were as follows:

pH 6.6 ± 0.1, fat 3.15 ± 0.20%, solids-non-fat (SNF) 8.40 ± 0.15%, density 1.0278 ± 0.0005 g/mL, protein content 3.20 ± 0.10%, added water 0.00%, freezing point—0.565 ± 0.005 °C, antibiotic test (MilkSafeTM 3BTS kindly provided by CHR Hansen, Denmark)—negative.

### 2.2. Starter Culture

Freeze-dried starter culture YOFLEX^®^ for stirred yoghurt (CHR Hansen, Denmark) containing *Streptococcus thermophilus* and *Lactobacillus delbrueckii* spp. bulgaricus was used for direct application in quantity according to producer requirements.

### 2.3. Oils

Chia oil ((AV) 2.16 mgKOH/g; peroxide value (PV)- 4.72 meq O_2_/kg; α-linolenic acid- 63.9 mg/100 g) was purchased from Balcho Agro product Ltd. Cod liver oil (AV- 0.53 mgKOH/g; PV- 0 meq O_2_/kg; EPA- 19.8 mg/100 g; DHA- 11.6 mg/100 g;) and Algae oil (AV- 0.87 mgKOH; PV- 0 meq O_2_/kg; EPA- 1.6 mg/100 g; DHA- 43.9 mg/100 g) were kindly donated by BASF SE. Essential oil from coriander was purchased from Eterika Ltd. (Plovdiv, Bulgaria).

### 2.4. Alginic Acid Sodium Salt for Anginate Beads

The low-viscosity alginic acid sodium salt was purchased from Sigma-Aldrich Chemie, Bulgaria.

The alginate beads were made with calcium dichloride (37%) purchased from Biokom Trendafilov Ltd., Bulgaria.

All solvents were purchased from (Fillab Ltd., Plovdiv, Bulgaria). All analyses were performed using standard laboratory equipment available at the University of Food Technologies, Plovdiv, Bulgaria. All chemicals and reagents used in this study were of analytical grade.

### 2.5. Preparation of the Alginate Beads

Alginate beads were prepared using 2% (*w*/*v*) sodium alginate solution. The solution was obtained by dissolving sodium alginate in distilled water under continuous stirring. The alginate solution was heated to 70 °C and maintained at this temperature for 10 min. Subsequently, the mixture was homogenized for 2 min using an IKA^®^ T-18 Digital Ultra-Turrax^®^ laboratory homogenizer (Staufen, Germany) at 10,000 rpm. After homogenization, the alginate solution was cooled to room temperature. Following cooling, oil was incorporated into the alginate solution. The oil-to-alginate ratio was set at 25% (*w*/*w*). Three different oils were used as sources of omega-3 fatty acids: chia oil, cod liver oil, and algal oil. Coriander essential oil was added to each oil formulation to improve the sensory profile and provide antioxidant protection, based on preliminary experimental trials. Each oil was homogenized for 2 min at 18,000 rpm using the same homogenizer to obtain a uniform oil–alginate dispersion. The resulting emulsions were then extruded dropwise using a syringe into 2% (*w*/*v*) calcium dichloride solution. The beads were allowed to harden in the calcium solution, after which they were collected and rinsed with distilled water and kept at 4 ± 2 °C. The obtained alginate beads were subsequently used for yogurt fortification.

### 2.6. Preparation of Stirred Yoghurt

Stirred yogurt was produced from a mixture of milk standardized for fat content to 2% by separation in lines. The milk mixture was homogenized at 60–65 °C under a pressure of 14–18 MPa, followed by pasteurization at 92–95 °C with a holding time of 10–15 min. After heat treatment, the milk was cooled to the fermentation temperature of 44–45 °C. The cooled milk was transferred into double-walled fermentation tanks, where starter culture was added and fermentation was allowed to proceed until milk coagulation was achieved at pH 4.6. Upon reaching the target pH, the coagulated milk was simultaneously stirred and cooled until a temperature of 18–20 °C was attained. The cooled yogurt was then filled into polystyrene cups, and to each cup were added alginate beads containing encapsulated oils (1-control sample, 2-yoghurt with chia oil (0.63%), 3-yoghurt with algae oil (1.10%), 4-yoghurt with cod liver oil (1.55%)). Subsequently, the fortified yogurt was further cooled to 2–4 °C and stored under refrigerated conditions for 14 days. The selected concentrations were designed to contribute to recommended omega-3 intake levels, including EPA, DHA, and α-linolenic acid (ALA), in accordance with Regulation (EC) No 1924/2006 and the Bulgarian Ordinance No. 1 on physiological norms of nutrition and EFSA recommendations (250 mg/day EPA + DHA and 0.5% of total energy for ALA).

### 2.7. Determination of Titratable Acidity

The titratable acidity was determined according to the titrimetric method (BNS 1111-80) [9].

### 2.8. Determination of pH

pH was determined using a calibrated digital pH-meter WTW pH 7110 (InoLab, Weilheim, Germany).

### 2.9. Determination of Dry Matter

The dry matter was determined by the gravimetric method (ISO 5534:2004) [10].

### 2.10. Determination of Fat

The fat content of the yogurt samples was determined using the Rose–Gottlieb method according to ISO 1211:2010. [11]

The percentage of fat content was calculated according to Equation:

(1) % Fat = (weight of extracted fat/weight of sample) * 100

### 2.11. Water-Holding Capacity

The water-holding capacity (WHC) of the yogurt samples was evaluated by centrifugation. A total of 10 g of each sample was placed in centrifuge tubes and centrifuged at 4000 rpm for 30 min using an ST 802 A centrifuge. Following centrifugation, the separated supernatant was collected and weighed.

The WHC (%) was calculated using the equation:

(2) WHC = [(YW − SW)/YW] * 100

where:

WHP—Water-holding capacity (%);

YW—Yoghurt Weight (g);

SW—Supernatant Weight (g).

### 2.12. Antioxidant Activity

Extraction was performed by weighing 2.5–2.6 g of each sample into a 15 mL graduated Falcon tube with a cap. The volume was adjusted to 10 mL with 95% (*v*/*v*) ethanol. The samples were subjected to ultrasonic extraction for 15 min in an ultrasonic bath operating at 45 kHz at 30 °C. Subsequently, the extracts were centrifuged at 3000 rpm for 10 min, and the supernatant was used for the determination of antioxidant activity and total polyphenol content [12].

The DPPH radical scavenging activity was assessed using a reaction mixture consisting of 2.85 mL of DPPH reagent (2,2-diphenyl-1-picrylhydrazyl) (Sigma-Aldrich, Merck, Germany) and 0.15 mL of the analyzed sample. The mixture was incubated at 37 °C for 15 min. Absorbance was recorded at 517 nm against a methanol blank. The antioxidant activity was expressed as mM Trolox equivalents (TE) per gram of dry weight sample, as described by Ivanov et al. [12].

The FRAP reagent was freshly prepared by mixing 300 mM acetate buffer (pH 3.6), 10 mM 2,4,6-tris(2-pyridyl)-s-triazine (TPTZ) in 40 mM hydrochloric acid, and 20 mM iron (III) chloride hexahydrate (Sigma-Aldrich, Merck, Germany) dissolved in distilled water, in a volumetric ratio of 10:1:1. For the assay, 3 mL of the FRAP reagent was combined with 0.1 mL of the sample extract and incubated in the dark at 37 °C for 10 min. Absorbance was measured at 593 nm using distilled water as a blank. Results were expressed as mM Trolox equivalents (TE) per gram of dry weight sample according to the methodology detailed by Ivanov et al. [12].

The total phenolic content was evaluated using the Folin–Ciocalteu reagent. The analyzed sample (0.2 mL) was mixed with 1 mL Folin–Ciocalteu reagent diluted 1:4, and then 0.8 mL 7.5% Na_2_CO_3_ was added then measured after 20 min at 765 nm against a blank sample. The results were expressed in mg equivalent of gallic acid (GAE) per g of the sample [12].

### 2.13. Determination of Fat Acid Value and Peroxide Value

The fats were extracted from yoghurt samples according to the Bligh and Dyer’s (1959) using chloroform/methanol (1:1 *v*/*v*) [13].

Determination of acid value was made according to ISO 660:2020 [14]. The fats were dissolved in a solvent mixture, and the free fatty acids were quantified by titration with an ethanolic potassium hydroxide solution.

The acid value (AV) was calculated using the following formula:

(3) AV = V.F. 5.6104/m, mgKOH/g oil

where:

V = the volume of 0.1 n alcoholic potassium hydroxide in mL, consumed in the titration of the oil

F = the factor of 0.1 N alcoholic solution of potassium hydroxide

5.6104 = the volume of potassium hydroxide, in grams, contained in 1 mL of 0.1 N alcoholic potassium hydroxide solution

m = the weight in grams of the sample

Peroxide Value (PV) was determined according to Popa et al. [15] with modifications. PV was determined as 0.200 ± 0.0002 g of oil was dissolved with 5 mL chloroform and 2.5 mL acetic acid, after that the sample was stirred until the oil was completely dissolved. Subsequently, 1 mL of a 50% potassium iodide solution was added, and the mixture was allowed to stand in the dark for 5 min. Afterwards, 15–20 mL of distilled water and a few drops of a 1% starch solution were added, resulting in the appearance of a blue-violet coloration. The sample was titrated with 0.002 N sodium thiosulfate solution, under continuous homogenization by shaking, until the coloration in the aqueous (upper) layer disappeared. The volume of sodium thiosulfate solution consumed was recorded in mL. A blank sample was prepared under the same conditions, containing the reagents without the oil.

The peroxide value in meqO2/kg was calculated using the formula:

(4) PV = (V − V_0_).0.002.1000/m, meqO_2_/kg

where:

V = the volume in mL of 0.002 n sodium thiosulfate used to titrate the oil sample

V_0_ = the volume in mL of 0.002 n sodium thiosulfate used to titrate the blank sample

m = the weight in grams of the sample

### 2.14. Microbiological Analysis

Yogurt samples intended for microbiological examination were prepared in accordance with BNS EN ISO 6887-5:2020 [16]. The enumeration of Lactobacillus spp. was performed following the procedure described in BNS EN ISO 9232:2005 [17], while *Streptococcus* spp. were quantified according to BNS EN ISO 7889:2005 [18]. Viable counts were determined using selective synthetic culture media, namely MRS agar (Merck) for *Lactobacillus* spp. and M17 agar (Merck) for *Streptococcus* spp. Listeria monocytogenes was analyzed according to ISO 11290-1:2017 [19].

### 2.15. Total Fatty Acids, Polar Metabolites and Nonpolar Metabolites

Polar and non-polar metabolites were analyzed using gas chromatography–mass spectrometry (GC–MS) according to the method described in Amirdivani and Boba [20], with minor modifications for yogurt samples. Freeze-dried yogurt samples (100 mg) were extracted with 500 µL of methanol. Internal standards were added to the extraction mixture, including 50 µL of ribitol (1 mg/mL) for polar metabolites and 50 µL of nonadecanoic acid (1 mg/mL) for non-polar metabolites. The samples were vortexed for 10 s and incubated for 30 min at 70 °C under agitation (300 rpm).

After cooling, 300 µL of distilled water and 500 µL of chloroform were added to facilitate phase separation. The mixture was centrifuged at 13,000 rpm for 10 min at 22 °C, resulting in separation of the polar and non-polar fractions, which were processed independently.

For the analysis of polar metabolites, 300 µL of the upper phase was transferred to a clean vial and evaporated to dryness under vacuum. Derivatization was performed by adding 100 µL of methoxyamine hydrochloride solution in pyridine (20 mg/mL) followed by incubation at 70 °C for 90 min. Subsequently, silylation was carried out by adding 50 µL of N,O-bis(trimethylsilyl)trifluoroacetamide (BSTFA) and incubating at 70 °C for 30 min.

For fatty acid analysis, 300 µL of the lower phase was evaporated to dryness and subjected to transmethylation using 1 mL of 1 M sulfuric acid in methanol at 96 °C for 90 min. The resulting fatty acid methyl esters (FAMEs) were extracted with hexane (3 × 500 µL), evaporated, and derivatized using 50 µL BSTFA and 50 µL pyridine at 70 °C for 30 min.

GC–MS analysis was performed using an Agilent 7890A gas chromatograph coupled with a 5975C mass selective detector and equipped with an HP-5MS capillary column (30 m × 0.32 mm × 0.25 µm). Helium was used as the carrier gas at a flow rate of 1 mL/min. The injection volume was 1 µL with a split ratio of 20:1.

For polar metabolites, the oven temperature program started at 100 °C and was held for 2 min, followed by an increase to 180 °C at 15 °C/min (1 min hold), and finally increased to 300 °C at 5 °C/min with a final hold of 10 min. For fatty acid analysis, the temperature program began at 70 °C (1 min) and increased to 300 °C at 5 °C/min with a final hold of 10 min.

Injector and detector temperatures were maintained at 250 °C. Mass spectra were recorded in the range of *m*/*z* 50–550. Metabolite identification was performed by comparing retention times and mass spectra with reference libraries from the Golm Metabolome Database (GMD) and the National Institute of Standards and Technology (NIST 08) database [21].

### 2.16. Sensory Analysis

Sensory evaluation of the yogurt samples was performed in accordance with ISO 8589 [22] by a trained panel consisting of 20 assessors with experience in the sensory evaluation of dairy products. All panelists were adults (over 18 years of age), included both male and female participants, and had no prior experience with omega-3-enriched products. Sensory attributes were evaluated using a five-point hedonic scale (1–5), where 1 indicated the lowest and 5 the highest level of quality/acceptability. The sensory attributes evaluated included taste and flavour, texture, and colour. The evaluation was carried out under controlled environmental conditions with standardized lighting and temperature. The yogurt samples were coded using randomly generated three-digit numbers and presented to the panelists in randomized order to minimize potential bias. Between samples, panelists rinsed their mouths with water to avoid carry-over effects. The sensory results were expressed as mean values ± standard deviation. Statistical analysis was performed using analysis of variance (ANOVA), followed by post hoc multiple comparison tests to determine statistically significant differences among samples at a significance level of *p* < 0.05. In accordance with institutional and national guidelines, ethical approval was not required for sensory testing involving trained panelists. Nevertheless, all participants were informed about the study objectives and provided voluntary consent prior to participation.

### 2.17. Rheological Characteristics

The rheological properties of yogurt samples were analyzed using a HAAKE MARS rheometer (Thermo Scientific, Karlsruhe, Germany). All measurements were performed in triplicate, and the results were expressed as mean values. The temperature during the tests was maintained at 20 °C using a Peltier system. Dynamic oscillatory measurements were carried out using a parallel plate geometry (50 mm diameter, 1 mm gap). The storage modulus (G′) and loss modulus (G″) were obtained using the instrument software (RheoPlus/32 V2.62) for each sample.

The rheological behavior of yogurt samples was evaluated using both empirical and structural models in order to describe their flow and viscoelastic properties.

For the characterization of steady shear behavior, the Power Law (Ostwald–de Waele) model was applied:

(5) $$\tau =K\cdot {\dot{\gamma }}^{n}$$

where τ is the shear stress (Pa), K is the consistency coefficient (Pa·s^n^), and n is the flow behavior index. Values of n < 1 indicate shear-thinning (pseudoplastic) behavior, which is typical for fermented dairy systems.

To account for the presence of yield stress, the Herschel–Bulkley model was additionally used:

(6) $$\tau =\tau_{0}+K\cdot {\dot{\gamma }}^{n}$$

where τ_0_ represents the yield stress (Pa), corresponding to the minimum stress required to initiate flow. This model is particularly suitable for structured systems such as yogurt, where a three-dimensional gel network must be disrupted before flow occurs.

For samples containing chia and cod liver oil, more complex models were employed. The Cross model was used to describe viscosity as a function of shear rate:

(7) $$\eta =\eta_{\infty }+\frac{\eta_{0}-\eta_{\infty }}{1+(k\cdot \dot{\gamma }{)}^{m}}$$

where η_0_ is the zero-shear viscosity, reflecting the structural integrity of the system at rest.

Additionally, the Carreau–Yasuda model was applied to better describe the transition between Newtonian and non-Newtonian flow regions:

(8) $$\eta =\eta_{\infty }+\left(\eta_{0}-\eta_{\infty }\right){\left[1+(\lambda \cdot \dot{\gamma }{)}^{a}\right]}^{\frac{n-1}{a}}$$

where λ is a characteristic relaxation time, a is a dimensionless parameter describing the width of the transition region, and η∞ is the infinite-shear viscosity.

These models provide a comprehensive description of the yogurt structure, including its resistance to flow, structural breakdown under shear, and flow behavior across different shear conditions.

### 2.18. Statistical Analysis

Statistical analysis was carried out using one-way and two-way analysis of variance (ANOVA) performed in Microsoft Excel 2024. The results are expressed as mean values ± standard deviation (SD) based on four determinations (n = 4). Two independent yogurt production trials were conducted as biological replicates, and each sample was analyzed in duplicate as analytical replicates.

Before performing ANOVA, the assumptions of normal distribution and homogeneity of variances were verified using Cochran’s test. Differences between mean values were assessed using Fisher’s least significant difference (LSD) test combined with Scheffé’s procedure for multiple comparisons. Statistical significance was accepted at a level of α = 0.05 (*p* < 0.05). Significant differences between samples are indicated in the tables by different superscript letters.

## 3. Results

### 3.1. Physicochemical Characteristics

Table 1 summarizes the physicochemical characteristics of the yogurts during storage. The incorporation of alginate-encapsulated omega-3 oils did not significantly affect (*p* < 0.05) titratable acidity at the beginning of storage. During refrigerated storage, slight changes in pH and titratable acidity were observed across all treatments. Lactic acid content showed minor variations over time without marked differences between control and enriched samples.

Total solids varied depending on oil type, while storage time had limited influence on this parameter. Water-holding capacity decreased during storage in all samples, with no consistent enhancement observed in enriched formulations. Fat content was higher in omega-3-enriched yogurts compared to the control and remained stable throughout storage.

### 3.2. Oxidative Stability

The oxidative stability parameters are presented in Table 2. Acid value differed between control and omega-3-enriched yogurts. The control exhibited higher initial values, while enriched samples showed lower initial acid values. During refrigerated storage, acid value increased in all enriched formulations.

Peroxide value clearly distinguished control from enriched samples. No detectable (*p* < 0.05) primary oxidation products were observed in the control during storage, whereas peroxide formation occurred in all omega-3-enriched yogurts. Peroxide value increased progressively during storage, with marine oil formulations showing more pronounced changes compared to plant-based enrichment.

### 3.3. Fatty Acid Composition, Trans Fatty Acid Composition and Polar Metabolites

**Figure 1:**
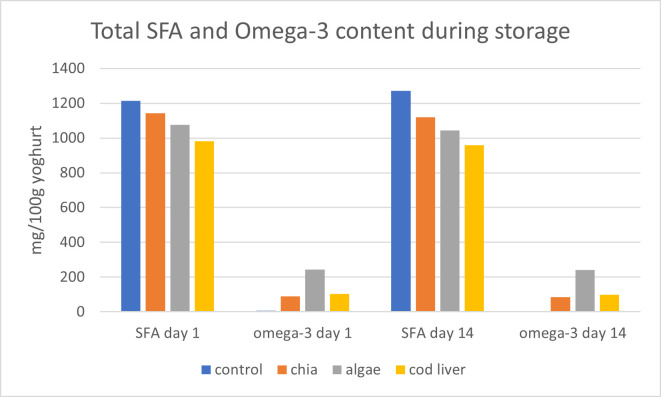
Total SFA and Omega-3 content during storage.

The fatty acid composition of the samples is summarized in Figure 1, while detailed profiles are provided in Table S1 (Supplementary Materials). To enable a more meaningful comparison between samples, selected fatty acid groups are also expressed on an absolute basis (mg/100 g yogurt), calculated from the total fat content. The incorporation of alginate-encapsulated omega-3 oils resulted in a substantial enrichment of total omega-3 fatty acids compared to the control sample. Yogurt enriched with chia oil showed higher levels of α-linolenic acid (ALA), whereas samples containing algal and cod liver oils exhibited the presence of long-chain omega-3 fatty acids (EPA and DHA), which were not detected in the control. These fatty acids remained relatively stable during refrigerated storage.

It should be noted that fatty acid composition expressed as relative percentages may be influenced by a dilution effect due to the constant-sum constraint; therefore, changes in the proportion of other fatty acids are interpreted cautiously and not as absolute increases or decreases. The ω-3:ω-6 ratio in enriched samples improved compared to the control, approaching nutritionally recommended values and indicating successful omega-3 enrichment. The estimated amount of yogurt required to meet the recommended daily intake of omega-3 fatty acids was calculated based on the experimentally determined fatty acid composition of the final products. The contents of EPA, DHA, and ALA were obtained from fatty acid analysis and expressed relative to the fat content of the yogurt per 100 g of product. The calculations represent approximate values and do not account for potential changes due to oxidation or matrix interactions.

Table 3 shows differences in the trans fatty acid composition of yogurts. The trans fatty acid profile of all samples was dominated by naturally occurring ruminant trans isomers, particularly trans-11 vaccenic acid. The control sample exhibited the highest proportion of total trans isomers. Omega-3 enrichment did not result in an increase in total trans fatty acid levels. Variations in the relative proportions of individual trans isomers among samples may reflect differences in fat source composition rather than absolute changes in their content. Storage did not result in noticeable changes in total trans fatty acids across treatments.

Table 4 presents the quantity of total polar metabolites in yogurts. Differences in amino acid composition were observed among treatments and during storage. Variations were particularly evident for several essential and branched-chain amino acids. Marine oil-enriched yogurts showed a redistribution of certain amino acids compared to the control, whereas chia-enriched samples displayed a profile closer to the control. Residual lactose decreased during storage in all treatments. Changes in monosaccharides and organic acids were also observed over time. Lactic acid remained the predominant organic acid in all samples. Storage time exerted a stronger influence than oil type on most organic acids.

### 3.4. Antioxidant Activity, Microbiological and Rheological Characteristics, Sensory Evaluation

The antioxidant activity of yogurt samples was evaluated during storage. The results are summarized in Table 5.

**Table 5:** Antioxidant activity of yoghurt.

Type of Analysis	Storage Day	Control Sample	Yoghurt with Chia Oil	Yogurt with Algae Oil	Yoghurt with Cod Liver Oil
DPPH method mM TE/100 g	1	45.11 ± 1.87 ^aA^	41.17 ± 2.32 ^aA^	43.93 ± 1.82 ^aA^	45.63 ± 0.41 ^aA^
	14	43.41 ± 1.47 ^aA^	39.78 ± 1.00 ^bA^	42.70 ± 1.78 ^aA^	37.06 ± 0.20 ^bB^
ABTS method mM TE/100 g	1	45.66 ± 1.13 ^aA^	47.09 ± 1.15 ^aA^	47.36 ± 1.12 ^aA^	44.33 ± 1.42 ^aA^
	14	29.72 ± 2.48 ^aB^	29.67 ± 0.83 ^aB^	27.51 ± 0.70 ^aB^	36.74 ± 1.00 ^bB^
Total polyphenols mg GAE/100 g	1	8.09 ± 0.57 ^aA^	7.07 ± 0.20 ^bA^	7.01 ± 0.13 ^bA^	6.72 ± 0.30 ^bA^
	14	7.71 ± 0.48 ^aA^	6.31 ± 0.40 ^bB^	6.42 ± 0.37 ^bB^	6.35 ± 0.11 ^bA^

a,b letters point out differences (*p* < 0.05) between yogurt samples. A,B letters point out differences (*p* < 0.05) between storage days.

Antioxidant capacity varied depending on the analytical method and storage time. At the initial stage, no substantial differences were observed among treatments. During storage, antioxidant activity decreased in all samples, with a more pronounced decline observed in ABTS values compared to DPPH.

Total polyphenol content showed slight reductions over storage in enriched samples. No drastic (*p* < 0.05)depletion of antioxidant-related parameters was detected.

**Figure 2:**
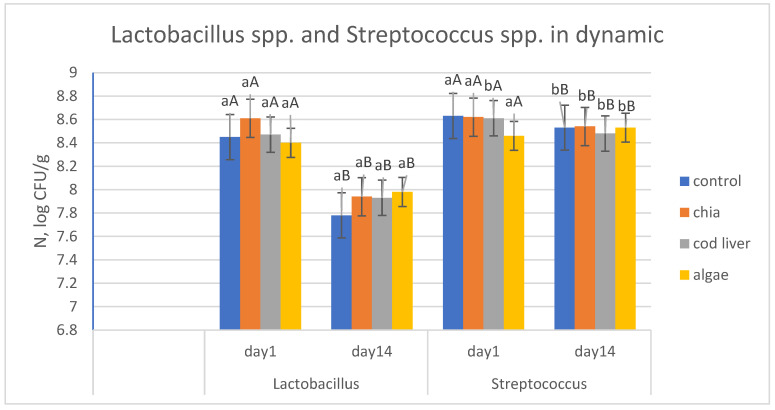
Microbiological profile of starter culture during storage. a,b letters point out differences (*p* < 0.05) between yogurt samples. A,B letters point out differences (*p* < 0.05) between storage days.

Figure 2 presents the growth dynamics of *Lactobacillus* spp. and *Streptococcus* spp. in stirred yogurts during 14 days of refrigerated storage. Both microorganisms remained viable throughout storage across all treatments. A slight reduction in viable counts was observed over time. No pronounced differences were detected between control and omega-3-enriched samples. All yogurt samples were analyzed for the presence of *Listeria monocytogenes*, and the pathogen was not detected, confirming the microbiological safety of the products.

**Figure 3:**
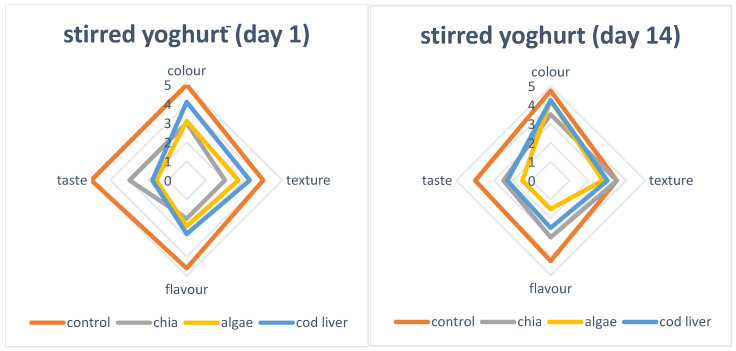
Sensory evaluetion of yoghurts during storage.

Figure 3 presents the sensory evaluation of yogurts at different storage times. At the initial stage, the control sample received the highest overall sensory scores, particularly for taste and flavour. Among enriched samples, chia oil yogurt showed scores closest to the control, while marine oil-enriched yogurts exhibited slightly lower flavour and taste ratings. Texture differences among treatments were moderate. After refrigerated storage, a general decline in flavour scores was observed across all treatments. Marine oil formulations showed a more pronounced reduction in flavour compared to chia oil yogurt. Texture attributes remained relatively stable during storage. The absence of volatile compound analysis (e.g., GC-MS) represents a limitation of the present study, as it does not allow detailed assessment of secondary oxidation products and their contribution to sensory quality.

Table 6 presents the main rheological parameters obtained from oscillatory measurements at a frequency of 1 Hz. In all samples, the storage modulus (G′) was higher than the loss modulus (G″), indicating predominantly elastic behavior. Differences between formulations were observed depending on the type of oil incorporated. The yogurt enriched with cod liver oil exhibited the highest storage modulus, while the chia and algal oil samples showed distinct but stable viscoelastic characteristics. During storage, all samples maintained their viscoelastic structure with only minor variations in the loss tangent (tan δ).

The flow behavior of the samples was investigated (Table 7 and Table 8). The Power Law and Herschel–Bulkley models were applicable to the control and algal oil-enriched samples, as they adequately describe simple pseudoplastic fluids with proteins integrated into the milk matrix. However, these models were not able to capture the disentanglement of long-chain structures associated with chia. This behavior was better described by the Cross and Carreau–Yasuda models. The use of these models was necessary to account for the nonlinear viscosity behavior over a wide range of shear rates, which is typical of complex food systems containing hydrocolloids (chia) and lipids (cod liver oil) that form structured microenvironments.

## 4. Discussion

### 4.1. Physicochemical Characteristics

The absence of significant differences in titratable acidity and pH between control and enriched samples indicates that alginate-encapsulated omega-3 oils did not interfere with starter culture activity. Similar findings were reported by Tamjidi et al. [4], who observed no significant differences in pH or titratable acidity in yogurt enriched with microencapsulated fish oil. In both cases, the gradual decrease in pH during storage reflects continued metabolic activity of starter cultures rather than lipid-induced alterations. Minor variations in lactic acid content further confirm that encapsulated oils neither inhibited nor overstimulated lactic acid bacteria. The preservation of fermentation dynamics suggests that encapsulation limited direct interactions between polyunsaturated fatty acids and microbial cells. Although total solids differed slightly among treatments, storage time had minimal impact, indicating overall structural stability. Unlike Tamjidi et al. [4], who reported improved water-holding capacity in yogurts enriched with gelatin–gum arabic microcapsules, the present study did not show a similar enhancement. This discrepancy may be attributed to differences in encapsulating materials, as alginate beads likely interact differently with the casein network and water-binding mechanisms. The increase in fat content in enriched yogurts is consistent with the addition of encapsulated oils. Comparable trends were described by Yusuf et al. [23] in omega-3–fortified Greek-type yogurt. The stability of fat levels during storage suggests effective encapsulation and minimal lipid leakage, while variations among oil types likely reflect differences in encapsulation efficiency rather than matrix instability.

### 4.2. Oxidative Stability

The higher initial acid value in the control reflects the intrinsic presence of free fatty acids in milk fat, whereas the lower initial values in enriched samples indicate that encapsulated oils did not promote immediate hydrolytic degradation. The gradual increase in acid value during storage is consistent with progressive lipid hydrolysis in systems containing polyunsaturated fatty acids. Similar trends were reported by Moghadam et al. [6], who observed limited but measurable hydrolysis in fish oil–fortified fermented milk.

Peroxide formation in enriched samples confirms their increased susceptibility to primary oxidation due to higher unsaturation levels. In agreement with Tamjidi et al. [4], peroxide values increased during storage in fish oil-fortified yogurt while remaining within acceptable limits. The more pronounced oxidation in marine oil formulations is attributable to the greater oxidative sensitivity of EPA- and DHA-rich lipids compared to α-linolenic acid–rich oils. Nevertheless, the controlled increase observed in this study suggests that alginate encapsulation effectively limited oxygen diffusion and slowed oxidative propagation. Similar protective effects of encapsulation systems have been described by Moghadam et al. [6].

### 4.3. Fatty Acid Composition, Trans Fatty Acid Composition and Polar Metabolites

Changes in fatty acid distribution should be interpreted with caution, as the use of relative percentage data introduces a constant-sum constraint. Therefore, apparent variations in the proportion of specific fatty acids may reflect a dilution effect associated with the incorporation of non-dairy lipid sources rather than absolute changes in their concentration.

The observed enrichment with omega-3 fatty acids, particularly α-linolenic acid in chia-enriched samples and EPA and DHA in marine oil formulations, confirms the successful incorporation of these bioactive lipids into the yogurt matrix. Similar modifications in fatty acid profiles following oil incorporation have been reported by Moghadam et al. [6], Kibui [8], Derewiaka et al. [7], and Zhong et al. [5].

The stability of EPA and DHA during storage suggests that alginate encapsulation provided protection against rapid oxidative degradation. In combination with controlled peroxide formation, this supports the effectiveness of the encapsulation approach in maintaining lipid stability.

Overall, omega-3 enrichment modified the fatty acid profile of yogurt by increasing the relative contribution of polyunsaturated fatty acids, without evidence of instability during storage.

The predominance of trans-11 vaccenic acid reflects the natural occurrence of ruminant-derived trans fatty acids formed during ruminal biohydrogenation [24]. The relative contribution of trans isomers in enriched samples is influenced by the proportion of dairy and non-dairy lipid sources, rather than indicating absolute changes in their concentration.

Importantly, no increase in total trans fatty acids was observed during storage, indicating that the applied technological conditions did not promote lipid isomerization. As emphasized by Jensen [25], trans fatty acid formation is typically associated with high-temperature processing or industrial hydrogenation rather than refrigerated storage conditions.

From a nutritional perspective, the detected trans fatty acids belong predominantly to naturally occurring dairy-derived isomers, which differ metabolically from industrial trans fats [26]. Therefore, omega-3 fortification via alginate encapsulation did not compromise lipid quality.

The observed differences in amino acid composition reflect ongoing proteolysis driven by the enzymatic system of yogurt starter cultures. Such dynamic changes are characteristic of fermented dairy products, where cell-envelope proteinases and intracellular peptidases continuously hydrolyze milk proteins during storage [27,28]. The redistribution of certain amino acids in marine oil-enriched samples suggests that oil type and encapsulation may subtly modulate metabolic activity rather than inhibit proteolytic pathways. The progressive reduction in lactose confirms continued bacterial metabolism and post-acidification during refrigeration, a well-established phenomenon in yogurt systems [29]. Minor differences between treatments may be attributed to microstructural effects of encapsulated oils influencing substrate diffusion and microbial microenvironments. Organic acid evolution further confirms active fermentation metabolism. The predominance of lactic acid indicates sustained starter activity, while variations in intermediates linked to pyruvate metabolism may reflect shifts in redox balance and metabolic flux distribution. Such metabolic adjustments under varying compositional conditions have been previously described in fermented dairy matrices [30]. The stronger effect of storage time compared to oil type highlights that post-fermentation dynamics remain the primary driver of metabolite changes.

### 4.4. Antioxidant Activity, Microbiological and Rheological Characteristics and Sensory Evaluation

The absence of significant initial differences suggests that omega-3 enrichment alone did not markedly modify radical-scavenging capacity, which is consistent with observations in fortified dairy systems where lipid addition does not inherently enhance antioxidant activity [5]. The progressive decline in antioxidant capacity during storage reflects ongoing oxidative processes within the yogurt matrix. The stronger reduction detected by the ABTS assay compared to DPPH can be explained by methodological differences between the two assays, as ABTS is more responsive to both hydrophilic and lipophilic antioxidants [31]. The relatively moderate changes observed in DPPH values therefore likely reflect method-dependent sensitivity rather than severe compositional deterioration. The slight decrease in total polyphenol content may be associated with oxidation reactions or protein–polyphenol interactions. Such interactions can reduce measurable phenolic content without completely eliminating antioxidant functionality, as discussed by Ozdal et al. [32]. Overall, despite storage-related changes, the antioxidant system remained relatively stable, indicating that omega-3 incorporation did not induce severe oxidative imbalance.

The slight reduction in viable counts during storage is characteristic of yogurt systems and is generally associated with progressive acid stress and nutrient limitation. Similar observations were reported by Moghadam et al. [6], who demonstrated high survival rates of lactic acid bacteria in probiotic fermented milk enriched with nanoencapsulated fish oil during refrigerated storage. Comparable findings were described by Derewiaka et al. [7], who reported that chia oil enrichment did not disrupt starter culture balance or post-acidification dynamics. The maintenance of viable populations within the typical range for fermented dairy products indicates that alginate-encapsulated omega-3 oils did not exert inhibitory effects on starter cultures. These results confirm that omega-3 fortification via alginate beads is microbiologically compatible with traditional yogurt fermentation systems.

The higher initial sensory scores of the control reflect the typical flavour profile of traditional stirred yogurt. The relatively good acceptability of chia-enriched yogurt is consistent with findings by Derewiaka et al. [7], who reported that chia oil incorporation improved fatty acid composition without causing significant sensory deterioration.

The slightly lower flavour scores in marine oil-enriched samples can be attributed to the characteristic sensory properties of long-chain omega-3 fatty acids. Similar flavour deviations in fish oil–fortified yogurt were reported by Zhong et al. [5] and Tamjidi et al. [4], who observed storage-related flavour changes associated with lipid oxidation. The greater susceptibility of EPA- and DHA-rich oils to oxidative reactions explains the more pronounced sensory decline in marine oil formulations. As discussed by Shahidi and Ambigaipalan [3], the degree of unsaturation plays a critical role in flavour stability in omega-3-enriched foods.

The moderate texture changes observed suggest that encapsulation helped preserve structural properties, consistent with reports on fish oil–fortified fermented milk systems [6]. Overall, while encapsulation reduced the impact of storage-related flavour deterioration, it did not completely eliminate sensory changes. Although the present study was based on a trained sensory panel, consumer acceptance is a critical factor for the successful market implementation of omega-3-enriched dairy products. Previous studies have reported that consumer perception of such products may be influenced by flavor changes associated with marine oils, as well as by health-related awareness and perceived benefits [4,5]. Therefore, further studies involving consumer panels are necessary to evaluate acceptance and purchase intent in real market conditions.

Rheological characterization is essential for understanding the structural and mechanical properties of yogurt, particularly in relation to its response to applied stress. Parameters such as apparent viscosity, storage modulus (G′), and loss modulus (G″) provide insight into the strength and stability of the gel network [33]. Oscillatory rheology, especially under small-amplitude conditions, is widely used to evaluate the integrity of yogurt gels without disrupting their internal structure. The predominance of G′ over G″ observed in all samples confirms the formation of a stable, elastic gel network typical of fermented dairy systems. The relatively low values of tan δ further support the solid-like behavior of the samples, indicating that elastic properties dominate over viscous ones. This behavior is associated with a well-developed casein network capable of retaining water and maintaining structural integrity during storage. The higher storage modulus observed in the cod liver oil-enriched yogurt suggests enhanced interactions within the protein–lipid matrix. This effect may be attributed to the presence of polyunsaturated fatty acids, which can influence the organization of casein micelles and strengthen the gel structure. Similar effects of lipid components on the mechanical properties of dairy gels have been reported by Romeih et al. [34], who demonstrated that fat incorporation can modify gel firmness and elasticity. The addition of algal components may further contribute to structural reinforcement due to their protein content, which can interact with milk proteins and promote the formation of a more complex gel network. Previous studies have shown that the incorporation of microalgae or their extracts can alter the dairy matrix and improve rheological properties through protein–protein interactions [35,36]. In the case of chia-enriched yogurt, the observed rheological behavior can be associated with the formation of a secondary network structure, likely due to the presence of hydrophilic polysaccharides. These compounds enhance water-binding capacity and contribute to gel stabilization. The role of chia-derived polysaccharides in improving rheological properties has been previously highlighted [37].

During storage, the maintenance of low tan δ values indicates that the elastic nature of the gels was preserved over time. The slight decrease observed in chia-containing samples may be related to further strengthening of the secondary network, while the cod liver oil sample maintained the most pronounced elastic character, consistent with its higher storage modulus.

The rheological behavior of yogurt is strongly influenced by its microstructure and composition. In the present study, the control and algal oil-enriched samples exhibited flow characteristics typical of pseudoplastic systems, which can be effectively described by the Power Law and Herschel–Bulkley models. These models are widely applied to fermented dairy systems, where the casein network governs flow behavior and results in shear-thinning properties [29,33]. However, in samples containing chia and cod liver oil, the rheological response became more complex. The presence of chia is associated with the formation of a secondary network structure due to its high content of hydrophilic polysaccharides. These macromolecules contribute to water binding and structural reinforcement, while also undergoing alignment and disentanglement under shear, leading to pronounced shear-dependent viscosity changes. Similar behavior has been reported for chia-based systems, where polysaccharide-rich fractions significantly influence rheological properties [37]. The use of Cross and Carreau–Yasuda models allowed a more accurate description of viscosity behavior across a broad range of shear rates. These models are particularly suitable for complex food systems exhibiting transitions between Newtonian and non-Newtonian flow regions and are commonly applied to products containing hydrocolloids and dispersed lipid phases [38,39]. The incorporation of cod liver oil further contributes to structural heterogeneity, as lipid droplets interact with the protein matrix and influence flow resistance. Lipid–protein interactions have been shown to modify the mechanical and rheological properties of dairy gels, affecting both viscosity and structural stability [24,34].

Overall, the requirement for more advanced rheological models in enriched samples reflects the formation of complex microstructures resulting from interactions between proteins, lipids, and polysaccharides. This behavior is characteristic of enriched dairy systems containing bioactive components and highlights the importance of selecting appropriate rheological models for accurate characterization.

This study has several limitations that should be acknowledged. The storage period was limited to 14 days, which may not fully reflect long-term product stability. In addition, no in vitro digestion or bioaccessibility analysis was performed, limiting the evaluation of the nutritional functionality of the encapsulated omega-3 fatty acids. Furthermore, the sensory analysis was conducted using a trained panel, which may not fully represent consumer preferences. Future studies should address these aspects to provide a more comprehensive evaluation of the developed products.

## 5. Conclusions

The present study demonstrated that incorporation of alginate-encapsulated omega-3 oils into stirred yogurt is a technologically feasible strategy for modifying the fatty acid profile without compromising product quality. Enrichment significantly modified the fatty acid composition, resulting in an increased proportion of polyunsaturated fatty acids and enhanced levels of α-linolenic acid, EPA, and DHA depending on the oil source. Importantly, these long-chain omega-3 fatty acids remained stable during refrigerated storage, confirming the protective role of alginate encapsulation.

Although omega-3 fortification increased susceptibility to lipid oxidation, hydrolytic and primary oxidative changes remained controlled throughout storage. Peroxide formation was more pronounced in marine oil formulations, reflecting the higher degree of unsaturation. However, it should be noted that the absence of volatile compound analysis (e.g., GC-MS) limits the assessment of secondary oxidation products and their potential impact on sensory quality. Physicochemical parameters, including pH, titratable acidity, total solids, water-holding capacity, and fat content, were not adversely affected by oil incorporation. The encapsulated systems did not disrupt fermentation dynamics, as confirmed by stable lactic acid production and sustained viability of *Lactobacillus* spp. and *Streptococcus* spp. throughout storage. Metabolite profiling further supported the preservation of fermentation functionality, with storage time exerting a stronger influence than oil type on amino acids, carbohydrates, and organic acids. Sensory evaluation indicated that enrichment influenced flavour attributes more than texture or colour, with marine oils exhibiting slightly greater flavour sensitivity during storage. Nevertheless, no severe sensory deterioration was observed, and overall product acceptability remained satisfactory. Approximately 350 g, 260 g, and 120 g of yogurt enriched with chia, cod liver, and algal oil, respectively, would be required to contribute to the recommended daily omega-3 intake, based on experimentally determined fatty acid composition. Overall, the developed products demonstrated potential as omega-3-enriched dairy foods with enhanced nutritional value.

Alginate encapsulation appears to be a promising strategy for omega-3 fortification of fermented dairy products; however, further research is required to better understand its efficiency, stability, and bioaccessibility. Future studies should focus on in vitro digestion models to assess the bioaccessibility and release kinetics of encapsulated omega-3 fatty acids.

The proposed formulations show potential for industrial application, as the encapsulation approach is compatible with dairy processing technologies. However, successful commercialization requires compliance with Regulation (EC) No 1924/2006 and further validation at industrial scale.

## Figures and Tables

**Table 1:** Physicochemical characteristics of yoghurts.

Type of Analysis	Storage Day	Control Sample	Yoghurt with Chia Oil	Yogurt with Algae Oil	Yoghurt with Cod Liver Oil
Acidity, °T	1	91 ± 1.00 ^aA^	92.33 ± 2.51 ^aA^	91.66 ± 1.52 ^aA^	92.33 ± 2.51 ^aA^
	14	91 ± 1.00 ^aA^	93.33 ± 1.52 ^aA^	91.66 ± 2.08 ^aA^	91 ± 1.00 ^aA^
Lactic acid, %	1	0.81 ± 0.009 ^aA^	0.83 ± 0.022 ^aA^	0.082 ± 0.013 ^aA^	0.83 ± 0.022 ^aA^
	14	0.81 ± 0.009 ^aA^	0.84 ± 0.013 ^bA^	0.82 ± 0.018 ^aA^	0.81 ± 0.009 ^aA^
pH	1	4.29 ± 0.060 ^aA^	4.32 ± 0.030 ^aA^	4.28 ± 0.020 ^aA^	4.28 ± 0.020 ^aA^
	14	4.17 ± 0.025 ^aB^	4.17 ± 0.02 ^aB^	4.26 ± 0.055 ^cB^	4.27 ± 0.025 ^bA^
Total solids, %	1	11.43 ± 0.056 ^aA^	11.45 ± 0.045 ^aA^	12.01 ± 0.065 ^cA^	11.05 ± 0.051 ^bA^
	14	11.44 ± 0.045 ^aA^	11.45 ± 0.050 ^aA^	11.88 ± 0.076 ^cA^	11.08 ± 0.076 ^bA^
Water-holding capacity, %	1	42.88 ± 0.070 ^aA^	42.84 ± 0.052 ^aA^	42.77 ± 0.24 ^cA^	42.00 ± 0.56 ^bA^
	14	41.83 ± 0.076 ^aB^	41.79 ± 0.040 ^aB^	41.62 ± 0.072 ^cB^	41.10 ± 0.10 ^bB^
Fat, %	1	2.43 ± 0.057 ^aA^	2.56 ± 0.057 ^bA^	2.76 ± 0.057 ^cA^	2.63 ± 0.058 ^bA^
	14	2.33 ± 0.057 ^aA^	2.43 ± 0.058 ^bB^	2.73 ± 0.052 ^dA^	2.56 ± 0.057 ^cA^

a–d letters point out differences (*p* < 0.05) between yogurt samples. A,B letters point out differences (*p* < 0.05) between storage days.

**Table 2:** Oxidative stability of yoghurts.

Type of Analysis	Storage Day	Control Sample	Yoghurt with Chia Oil	Yogurt with Algae Oil	Yoghurt with Cod Liver Oil
Acid value, mg KOH/g	1	4.40 ± 0.08 ^aA^	2.89 ± 0.04 ^bA^	3.06 ± 0.05 ^cA^	3.05 ± 0.05 ^cA^
	14	4.11 ± 0.04 ^aB^	3.49 ± 0.01 ^bB^	3.86 ± 0.04 ^dB^	3.77 ± 0.03 ^cB^
Peroxide value, meq O_2_/kg	1	0 ^aA^	4.06 ± 0.05 ^bA^	4.43 ± 0.05 ^dA^	5.85 ± 0.04 ^cA^
	14	0 ^aA^	4.62 ± 0.07 ^bB^	7.55 ± 0.06 ^dB^	9.21 ± 0.11 ^cB^

a–d letters point out differences (*p* < 0.05) between yogurt samples; A,B letters point out differences (*p* < 0.05) between storage days.

**Table 3:** Trans fatty acid composition of yoghurt.

Trans Fatty Acids, %	Storage Day	Control Sample	Yoghurt with Chia Oil	Yogurt with Algae Oil	Yoghurt with Cod Liver Oil
Methyl trans-6-Octadecenoate + Trans-9-elaidic acid methyl ester	1	0.51 ± 0.05 ^aA^	0.36 ± 0.04 ^bA^	0.38 ± 0.04 ^bA^	0.37 ± 0.04 ^bA^
	14	0.48 ± 0.04 ^aA^	0.34 ± 0.04 ^bA^	0.35 ± 0.03 ^bA^	0.36 ± 0.04 ^bA^
Trans-11-Vaccenic methyl ester	1	1.79 ± 0.18 ^aA^	1.82 ± 0.18 ^aA^	1.21 ± 0.11 ^bA^	1.14 ± 0.11 ^bA^
	14	1.75 ± 0.15 ^aA^	1.80 ± 0.18 ^aA^	1.19 ± 0.11 ^bA^	1.15 ± 0.10 ^bA^
Methyl 9(Z), 11(E).—Octadecadienoate	1	0.35 ± 0.04 ^aA^	0.37 ± 0.05 ^aA^	0.27 ± 0.04 ^aA^	0.33 ± 0.05 ^aA^
	14	0.37 ± 0.04 ^aA^	0.37 ± 0.04 ^aA^	0.27 ± 0.02 ^aA^	0.32 ± 0.05 ^aA^
Trans-9,12-Octadecadienoic acid methyl ester	1	0.04 ± 0.01 ^aA^	0.06 ± 0.02 ^aA^	0.02 ± 0.01 ^aA^	0.02 ± 0.01 ^aA^
	14	0.04 ± 0.01 ^aA^	0.06 ± 0.02 ^aA^	0.02 ± 0.01 ^aA^	0.02 ± 0.01 ^aA^
Cis-9, trans- 12-Octadecadienoic acid methyl ester	1	0.13 ± 0.04 ^aA^	0.13 ± 0.04 ^aA^	0.10 ± 0.03 ^aA^	0.10 ± 0.03 ^aA^
	14	0.10 ± 0.04 ^aA^	0.10 ± 0.04 ^aA^	0.09 ± 0.04 ^aA^	0.10 ± 0.04 ^aA^
Ttrans-9 cis-12, -Octadecadienoic acid methyl ester	1	0.10 ± 0.03 ^aA^	0.09 ± 0.03 ^aA^	0.06 ± 0.02 ^aA^	0.07 ± 0.02 ^aA^
	14	0.10 ± 0.03 ^aA^	0.09 ± 0.03 ^aA^	0.05 ± 0.02 ^aA^	0.07 ± 0.02 ^aA^
Ttrans-9, trans-12, trans-15—Octadecantrienoic acid methyl ester	1	0.17 ± 0.04 ^aA^	0.15 ± 0.04 ^aA^	0.13 ± 0.03 ^aA^	0.12 ± 0.04 ^aA^
	14	0.15 ± 0.04 ^aA^	0.12 ± 0.04 ^aA^	0.11 ± 0.04 ^aA^	0.10 ± 0.04 ^aA^
Ttrans-9, trans-12, cis-15—Octadecantrienoic acid methyl ester	1	0.02 ± 0.01 ^aA^	0.02 ± 0.01 ^aA^	≤0.01	0.02 ± 0.01 ^aA^
	14	0.02 ± 0.01 ^aA^	0.02 ± 0.01 ^aA^	≤0.01	0.02 ± 0.01 ^aA^
Ttrans-9, cis-12, trans-15 -Octadecantrienoic acid methyl ester	1	≤0.01	≤0.01	≤0.01	≤0.01
	14	≤0.01	≤0.01	≤0.01	≤0.01
Cis-9, trans-12, trans-15—Octadecantrienoic acid methyl ester	1	0.04 ± 0.02 ^aA^	0.04 ± 0.02 ^aA^	0.06 ± 0.02 ^aA^	0.06 ± 0.02 ^aA^
	14	0.04 ± 0.02 ^aA^	0.04 ± 0.02 ^aA^	0.06 ± 0.03 ^aA^	0.06 ± 0.02 ^aA^
Cis-9, cis- 12, trans-15 -Octadecantrienoic acid methyl ester	1	≤0.01	0.02 ± 0.01 ^aA^	≤0.01	≤0.01
	14	≤0.01	0.02 ± 0.01 ^aA^	≤0.01	≤0.01
Cis-9, trans-12, cis-15 -Octadecantrienoic acid methyl ester	1	≤0.01	≤0.01	≤0.01	≤0.01
	14	≤0.01	≤0.01	≤0.01	≤0.01
Trans-9, cis-12, cis-15 -Octadecantrienoic acid methyl ester	1	≤0.01	≤0.01	≤0.01	≤0.01
	14	≤0.01	≤0.01	≤0.01	≤0.01

a,b letters point out differences (*p* < 0.05) between yogurt samples; A letter points out differences (*p* < 0.05) between storage days.

**Table 4:** Total polar metabolites in yoghurts.

Metabolites, g/100g	Storage Day	Control Sample	Yoghurt with Chia Oil	Yogurt with Algae Oil	Yoghurt with Cod Liver Oil
*Amino* *acids, % of total polar metabolites*					
Alanine	1	2.50 ± 0.18 ^aA^	1.59 ± 0.09 ^bA^	4.7 ± 0.29 ^dA^	1.42 ± 0.07 ^cA^
	14	2.84 ± 0.20 ^aA^	1.61 ± 0.12 ^bA^	4.49 ± 0.37 ^dA^	1.25 ± 0.08 ^cB^
Glycine	1	1.06 ± 0.08 ^aA^	0.94 ± 0.05 ^aA^	0.67 ± 0.04 ^cA^	0.84 ± 0.04 ^bA^
	14	0.75 ± 0.05 ^aB^	0.95 ± 0.07 ^bA^	0.64 ± 0.05 ^aA^	0.74 ± 0.05 ^aB^
Valine	1	2.96 ± 0.22 ^aA^	2.61 ± 0.14 ^aA^	1.85 ± 0.11 ^cA^	2.32 ± 0.11 ^bA^
	14	2.09 ± 0.15 ^aB^	2.64 ± 0.20 ^bA^	1.77 ± 0.14 ^aA^	2.04 ± 0.13 ^aB^
Leucine	1	1.64 ± 0.12 ^aA^	4.97 ± 0.27 ^bA^	3.53 ± 0.22 ^dA^	4.43 ± 0.21 ^cA^
	14	3.99 ± 0.28 ^aB^	5.03 ± 0.37 ^bA^	3.37 ± 0.28 ^aA^	3.9 ± 0.26 ^aB^
Isoleucine	1	2.48 ± 0.18 ^aA^	2.19 ± 0.12 ^aA^	1.55 ± 0.09 ^cA^	1.95 ± 0.09 ^bA^
	14	1.76 ± 0.12 ^aB^	2.22 ± 0.16 ^bA^	1.72 ± 0.11 ^aB^	1.48 ± 0.12 ^cA^
Proline	1	6.14 ± 0.45 ^aA^	4.4 ± 0.24 ^bA^	3.84 ± 0.23 ^cA^	3.91 ± 0.19 ^cA^
	14	4.34 ± 0.30 ^aB^	4.45 ± 0.33 ^aA^	3.67 ± 0.30 ^bA^	3.44 ± 0.23 ^bB^
Serine	1	3.86 ± 0.28 ^aA^	3.37 ± 0.19 ^bA^	2.41 ± 0.15 ^dA^	3 ± 0.14 ^cA^
	14	2.73 ± 0.19 ^aB^	3.41 ± 0.25 ^bA^	2.31 ± 0.19 ^aA^	2.64 ± 0.17 ^aB^
Threonine	1	3.29 ± 0.24 ^aA^	2.9 ± 0.16 ^aA^	2 ± 0.12 ^cA^	2.58 ± 0.12 ^bA^
	14	2.33 ± 0.16 ^aB^	2.94 ± 0.22 ^aA^	1.91 ± 0.16 ^bA^	2.27 ± 0.15 ^aB^
Aspartic acid	1	4.96 ± 0.36 ^aA^	2.42 ± 0.13 ^bA^	5.29 ± 0.32 ^aA^	2.16 ± 0.10 ^cA^
	14	3.51 ± 0.25 ^aB^	2.45 ± 0.18 ^bA^	5.05 ± 0.41 ^dA^	1.9 ± 0.13 ^cB^
Methionine	1	1.02 ± 0.07 ^aA^	1.82 ± 0.10 ^bA^	1.29 ± 0.08 ^dA^	1.62 ± 0.08 ^cA^
	14	1.43 ± 0.10 ^aB^	1.84 ± 0.14 ^bA^	1.23 ± 0.10 ^aA^	1.43 ± 0.09 ^aB^
Pyroglutamic acid	1	7.47 ± 0.55 ^aA^	3.97 ± 0.22 ^bA^	6.91 ± 0.42 ^aA^	3.53 ± 0.17 ^cA^
	14	5.28 ± 0.37 ^aB^	4.02 ± 0.30 ^bA^	6.6 ± 0.54 ^dA^	3.11 ± 0.21 ^cB^
Creatinine	1	2.34 ± 0.17 ^aA^	1.97 ± 0.11 ^bA^	3.34 ± 0.20 ^dA^	1.75 ± 0.08 ^cA^
	14	3.78 ± 0.26 ^aB^	1.99 ± 0.15 ^bA^	3.19 ± 0.26 ^dA^	1.54 ± 0.10 ^cB^
Arginine	1	0.66 ± 0.05 ^aA^	0.64 ± 0.04 ^aA^	0.53 ± 0.03 ^aA^	0.57 ± 0.03 ^aA^
	14	0.42 ± 0.03 ^aB^	0.66 ± 0.05 ^bA^	0.51 ± 0.04 ^cA^	0.5 ± 0.03 ^cB^
Glutamic acid	1	4.36 ± 0.32 ^aA^	2.28 ± 0.13 ^bA^	3.6 ± 0.22 ^dA^	2.03 ± 0.10 ^cA^
	14	3.08 ± 0.22 ^aB^	2.3 ± 0.17 ^bA^	3.44 ± 0.28 ^aA^	1.78 ± 0.12 ^cB^
Phenylalanine	1	1.80 ± 0.13 ^aA^	1.59 ± 0.09 ^bA^	1.12 ± 0.07 ^dA^	1.42 ± 0.07 ^cA^
	14	1.27 ± 0.09 ^aB^	1.61 ± 0.12 ^bA^	1.07 ± 0.09 ^cA^	1.25 ± 0.08 ^aB^
Lysine	1	1.71 ± 0.13 ^aA^	1.51 ± 0.08 ^aA^	1.07 ± 0.07 ^cA^	1.35 ± 0.06 ^bA^
	14	1.21 ± 0.08 ^aB^	1.53 ± 0.11 ^bA^	1.02 ± 0.08 ^cA^	1.18 ± 0.08 ^cB^
Tyrosine	1	0.70 ± 0.05 ^aA^	0.62 ± 0.03 ^aA^	0.44 ± 0.03 ^bA^	0.55 ± 0.03 ^aA^
	14	0.5 ± 0.03 ^aB^	0.63 ± 0.05 ^bA^	0.42 ± 0.03 ^aA^	0.48 ± 0.03 ^aB^
*Carbohydrates, % of total polar metabolites*					
Galactose	1	3.31 ± 0.24 ^aA^	2.84 ± 0.16 ^bA^	2.64 ± 0.16 ^bA^	3.21 ± 0.15 ^aA^
	14	2.09 ± 0.15 ^aB^	1.86 ± 0.14 ^aA^	2.57 ± 0.21 ^bA^	2.82 ± 0.19 ^bB^
Glucose	1	1.63 ± 0.12 ^aA^	1.72 ± 0.09 ^aA^	1.35 ± 0.08 ^cA^	1.95 ± 0.09 ^bA^
	14	1.04 ± 0.07 ^aB^	0.86 ± 0.06 ^bB^	0.71 ± 0.06 ^dB^	1.71 ± 0.11 ^cB^
Lactose	1	5.72 ± 0.42 ^aA^	6.89 ± 0.38 ^bA^	7.91 ± 0.48 ^cA^	7.79 ± 0.37 ^cA^
	14	5.53 ± 0.39 ^aA^	3.96 ± 0.29 ^bB^	4.3 ± 0.35 ^bB^	6.85 ± 0.45 ^cB^
*Organic acids, % of total polar metabolites*					
Pyruvac acid	1	3.4 ± 0.25 ^aA^	4.14 ± 0.23 ^bA^	3.24 ± 0.20 ^dA^	4.68 ± 0.22 ^cA^
	14	4.2 ± 0.29 ^aB^	5.11 ± 0.38 ^bA^	4 ± 0.33 ^aB^	5.78 ± 0.38 ^bB^
Lactic acid	1	8.36 ± 0.61 ^aA^	9.36 ± 0.51 ^bA^	10.55 ± 0.64 ^cA^	11.71 ± 0.56 ^cA^
	14	10.32 ± 0.72 ^aB^	7.55 ± 0.56 ^bB^	9.02 ± 0.74 ^aB^	7.44 ± 0.49 ^bB^
Oxalic acid	1	4.18 ± 0.30 ^aA^	4.97 ± 0.27 ^bA^	3.89 ± 0.24 ^aA^	5.61 ± 0.27 ^cA^
	14	5.33 ± 0.37 ^aB^	6.13 ± 0.45 ^aB^	7.59 ± 0.50 ^bB^	4.8 ± 0.39 ^aB^
Succinic acid	1	1.99 ± 0.15 ^aA^	2.43 ± 0.13 ^bA^	1.9 ± 0.12 ^aA^	2.74 ± 0.13 ^cA^
	14	2.46 ± 0.17 ^aB^	3 ± 0.22 ^bB^	2.35 ± 0.19 ^aB^	3.44 ± 0.23 ^bB^
Itaconic acid	1	3 ± 0.22 ^aA^	3.62 ± 0.20 ^bA^	2.83 ± 0.17 ^aA^	2.26 ± 0.11 ^cA^
	14	3.7 ± 0.26 ^aB^	4.46 ± 0.33 ^bB^	3.5 ± 0.29 ^aB^	2.79 ± 0.18 ^cB^
Fumaric acid	1	1.4 ± 0.10 ^aA^	1.71 ± 0.09 ^bA^	1.34 ± 0.08 ^aA^	1.93 ± 0.09 ^cA^
	14	1.73 ± 0.12 ^aB^	2.11 ± 0.16 ^bB^	1.65 ± 0.14 ^aB^	2.38 ± 0.16 ^bB^
Malic acid	1	4.15 ± 0.30 ^aA^	6.19 ± 0.34 ^bA^	4.43 ± 0.27 ^dA^	3.37 ± 0.16 ^cA^
	14	5.12 ± 0.36 ^aB^	5.94 ± 0.44 ^bA^	5.46 ± 0.45 ^aB^	4.16 ± 0.27 ^cB^
Fumaric acid	1	1.68 ± 0.12 ^aA^	2.03 ± 0.15 ^bA^	1.16 ± 0.12 ^dA^	2.3 ± 0.11 ^cA^
	14	2.07 ± 0.14 ^aB^	2.51 ± 0.19 ^bB^	1.43 ± 0.12 ^cB^	2.84 ± 0.19 ^bB^
Adipic acid	1	2.37 ± 0.17 ^aA^	3.05 ± 0.17 ^bA^	2.94 ± 0.18 ^dA^	3.55 ± 0.17 ^cA^
	14	2.92 ± 0.20 ^aB^	1.77 ± 0.13 ^bB^	3.63 ± 0.30 ^dB^	4.38 ± 0.29 ^cB^
α-Hydroxyglutaric acid	1	2.23 ± 0.16 ^aA^	1.81 ± 0.10 ^bA^	2.47 ± 0.15 ^aA^	3.36 ± 0.16 ^cA^
	14	2.75 ± 0.19 ^aB^	2.84 ± 0.27 ^aB^	3.04 ± 0.25 ^aB^	4.14 ± 0.27 ^bB^
Suberic acid	1	0.72 ± 0.05 ^aA^	1.33 ± 0.17 ^bA^	1.04 ± 0.06 ^cA^	1.5 ± 0.11 ^bA^
	14	0.89 ± 0.09 ^aB^	1.64 ± 0.12 ^bB^	1.29 ± 0.17 ^cB^	1.86 ± 0.18 ^bB^
Azelaic acid	1	2.49 ± 0.18 ^aA^	2.99 ± 0.16 ^bA^	2.36 ± 0.14 ^aA^	3.38 ± 0.16 ^cA^
	14	3.07 ± 0.21 ^aB^	3.69 ± 0.27 ^bB^	2.91 ± 0.24 ^aB^	4.17 ± 0.28 ^bB^
Citric acid	1	4.43 ± 0.32 ^aA^	5.11 ± 0.28 ^bA^	5.8 ± 0.35 ^bA^	5.25 ± 0.25 ^bA^
	14	5.47 ± 0.38 ^aB^	6.31 ± 0.47 ^aB^	7.17 ± 0.59 ^bB^	6.48 ± 0.43 ^bB^

a–d letters point out differences (*p* < 0.05) between yogurt samples. A,B letters point out differences (*p* < 0.05) between storage days.

**Table 6:** Main Rheological Parameters from Oscillatory Tests at a Frequency of 1 Hz.

Type of Analysis	Storage Day	Control Sample	Yoghurt with Chia Oil	Yogurt with Algae Oil	Yoghurt with Cod Liver Oil
G, Pa	1	46.01 ± 2.12 ^aA^	57.35 ± 3.28 ^bA^	60.15 ± 3.16 ^bA^	77.59 ± 5.15 ^cA^
	14	38.33 ± 1.22 ^aB^	62.72 ± 3.54 ^bA^	41.96 ± 2.52 ^aB^	55.51 ± 1.76 ^cB^
G″, Pa	1	14.84 ± 1.15 ^aA^	18.00 ± 1.16 ^bA^	20.22 ± 2.55 ^bA^	23.97 ± 2.10 ^bA^
	14	12.26 ± 1.34 ^aB^	19.51 ± 1.12 ^bA^	13.66 ± 2.14 ^aB^	15.74 ± 0.94 ^cB^
|η*|, Pa·s	1	7.69 ± 0.34 ^aA^	9.57 ± 0.35 ^bA^	10.10 ± 0.26 ^bA^	12.92 ± 0.28 ^cA^
	14	6.40 ± 0.23 ^aB^	10.45 ± 0.21 ^bB^	7.02 ± 0.32 ^cB^	8.88 ± 0.12 ^dB^
τ, Pa	1	0.59 ± 0.10 ^aA^	0.77 ± 0.23 ^aA^	0.82 ± 0.45 ^aA^	1.10 ± 0.98 ^aA^
	14	0.45 ± 0.12 ^aA^	0.86 ± 0.12 ^bA^	0.52 ± 0.19 ^aA^	0.70 ± 0.10 ^abA^
tg δ	1	0.322 ± 0.050 ^aA^	0.314 ± 0.054 ^aA^	0.336 ± 0.054 ^aA^	0.309 ± 0.054 ^aA^
	14	0.320 ± 0.021 ^aA^	0.311 ± 0.027 ^aA^	0.326 ± 0.025 ^aA^	0.284 ± 0.011 ^aA^

a–d letters point out differences (*p* < 0.05) between yogurt samples. A,B letters point out differences (*p* < 0.05) between storage days.

**Table 7:** Rheological Model Parameters of Control Yogurt and Yogurt Enriched with Algal Oil.

Type of Analysis	Storage Day	Control Sample	Yogurt with Algae Oil
Power Law Model			
K, Pa.sn	1	11.25 ± 0.25 ^aA^	10.95 ± 0.16 ^aA^
	14	9.70 ± 0.55 ^aB^	13.82 ± 1.22 ^bB^
n	1	0.252 ± 0.004 ^aA^	0.195 ± 0.005 ^bA^
	14	0.229 ± 0.001 ^aB^	0.150 ± 0.001 ^bB^
R2	1	0.983	0.969
	14	0.869	0.560
Herschel–Bulkley Model			
T0, Pa	1	7.03 ± 2.51 ^aA^	21.89 ± 0.15 ^bA^
	14	6.52 ± 0.70 ^aA^	23.11 ± 0.83 ^bB^
K, Pa.sn	1	7.60 ± 1.53 ^aA^	0.026 ± 0.034 ^bA^
	14	7.62 ± 0.51 ^aA^	0.01 ± 0.001 ^bA^
n	1	0.292 ± 0.024 ^aA^	1.060 ± 0.01 ^bA^
	14	0.234 ± 0.073 ^aA^	1.215 ± 0.167 ^bA^
R	1	0.979	0.994
	14	0.845	0.820

a,b letters point out differences (*p* < 0.05) between yogurt samples. A,B letters point out differences (*p* < 0.05) between storage days.

**Table 8:** Rheological Model Parameters of Yogurt Enriched with Chia Oil and Cod Liver Oil.

Type of Analysis	Storage Day	Yoghurt with Chia Oil	Yoghurt with Cod Liver Oil
Cross Model			
$\eta_{0}$, Pa	1	131.1 ± 2.5 ^a^	149.55 ± 12.52 ^b^
	14	95.54 ± 2.75 ^a^	300.39 ± 7.75 ^b^
$\eta_{\infty }$, Pa	1	45.20 ± 0.30 ^a^	38.62 ± 0.37 ^b^
	14	25.72 ± 0.32 ^a^	25.50 ± 0.55 ^a^
K, s	1	6.06 ± 0.69 ^a^	4.47 ± 0.71 ^b^
	14	3.09 ± 0.27 ^a^	2.31 ± 0.08 ^b^
n	1	0.06 ± 0.001 ^a^	0.06 ± 0.004 ^a^
	14	0.05 ± 0.001 ^a^	0.03 ± 0.008 ^b^
R	1	0.955	0.905
	14	0.946	0.994
Carreau-Yasuda Model			
$\eta_{0}$, Pa	1	113.41 ± 1.19 ^A^	The model is not applicable
	14	85.19 ± 1.19 ^aB^	268.41 ± 24.44 ^b^
$\eta_{\infty }$, Pa	1	45.90 ± 0.12 ^A^	The model is not applicable
	14	24.89 ± 1.20 ^aB^	15.43 ± 4.25 ^b^
K, s	1	0.09 ± 0.001 ^A^	The model is not applicable
	14	0.08 ± 0.001 ^aA^	0.05 ± 0.005 ^b^
n	1	0 ^A^	The model is not applicable
	14	0 ^aA^	0 ^a^
a	1	24,657 ± 20 ^A^	The model is not applicable
	14	309.39 ± 5.20 ^aB^	7.77 ± 0.83 ^b^
R	1	0.774	The model is not applicable
	14	0.879	0.946

a,b letters point out differences (*p* < 0.05) between yogurt samples. A,B letters point out differences (*p* < 0.05) between storage days.

## Data Availability

The data presented in this study are available on request from the corresponding author.

## Supplementary Materials

The following supporting information can be downloaded at: https://www.mdpi.com/article/10.3390/foods15091460/s1, Table S1: Fatty acid composition of yogurt.

## Abbreviations

The following abbreviations are used in this manuscript:

TPTZ	2,4,6-tris(2-pyridyl)-s-triazine
GAE	gallic acid
PV	peroxide value
FAMEs	fatty acid methyl esters
BSTFA	N,O-bis(trimethylsilyl)trifluoroacetamide
GC-MS	gas chromatography–mass spectrometry
DPPH	2,2-diphenyl-1-picrylhydrazyl (DPPH)
ABTS	2,2′-azino-bis(3-ethylbenzothiazoline-6-sulfonic acid)
DHA	docosahexaenoic acid
EPA	eicosapentaenoic acid
