# Supplementary material for: Yogurt Enriched with Omega-3 Fatty Acids
Source: Foods. 2026 Apr 22;15(9):1460. doi: 10.3390/foods15091460 (PMC13163698; doi:10.3390/foods15091460)
Supplement: Supplementary file 1 [file foods-15-01460-s001.zip › foods-4259360-supplementary.pdf]

Table S1. Fatty acid composition of yoghurts

Fatty acids, %	Storage day	Control sample	Yoghurt with chia oil	Yogurt with algae oil	Yoghurt with cod liver oil
Short and medium chain fatty acids					
Butiric acid, 4:0	1	1,91±0,18^{aA}	1,33±0,14^{bA}	1,41±0,15^{bA}	1,25±0,14^{bA}
	14	2,01±0,18^{aA}	1,30±0,14^{bA}	1,30±0,15^{bA}	1,15±0,14^{bA}
Caproic acid, 6:0	1	1,40±0,13^{aA}	1,06±0,09^{bA}	1,06±0,09^{bA}	0,95±0,08^{bA}
	14	1,60±0,13^{aA}	1,00±0,09^{bA}	1,0±0,09^{bA}	0,90±0,08^{bA}
Caprylic acid, 8:0	1	0,92±0,08^{aA}	0,73±0,07^{bA}	0,71±0,07^{bA}	0,64±0,06^{bA}
	14	0,95±0,08^{aA}	0,70±0,07^{bA}	0,67±0,07^{cA}	0,59±0,06^{cA}
Capric acid, 10:0	1	2,24±0,23^{aA}	1,88±0,21^{aA}	1,76±0,26^{aA}	1,58±0,32^{aA}
	14	2,56±0,23^{aA}	1,85±0,21^{bA}	1,62±0,26^{bA}	1,45±0,32^{bA}
Lauric acid, 12:0	1	2,76±0,27^{aA}	2,47±0,23^{aA}	2,31±0,23^{aA}	2,00±0,23^{aA}
	14	2,96±0,27^{aA}	2,35±0,23^{aA}	2,25±0,23^{aA}	2,00±0,23^{bA}
Long chain fatty acids					
Myristic acid, 14:0	1	9,28±0,91^{aA}	8,69±0,83^{aA}	9,18±0,87^{aA}	7,88±0,73^{aA}
	14	9,85±0,91^{aA}	8,25±0,83^{aA}	9,0±0,87^{aA}	7,73±0,73^{aA}
Palmitic acid, 16:0	1	34,63±1,78^{aA}	33,37±1,67^{aA}	31,20±1,88^{aA}	28,41±1,73^{bA}
	14	35,63±1,78^{aA}	33,12±1,67^{bA}	30,25±1,88^{bA}	27,95±1,73^{cA}
Stearic acid, 18:0	1	7,32±0,73^{aA}	7,43±0,67^{aA}	6,03±0,61^{aA}	6,19±0,67^{aA}
	14	7,85±0,73^{aA}	7,25±0,67^{aA}	6,0±0,61^{aA}	6,0±0,67^{aA}
Arachidonic acid, 20:0	1	0,10±0,03^{aA}	0,13±0,03^{aA}	0,09±0,03^{aA}	0,10±0,03^{aA}
	14	0,10±0,03^{aA}	0,10±0,03^{aA}	0,07±0,03^{aA}	0,10±0,03^{aA}
Behenic acid, 22:0	1	0,05±0,02^{aA}	0,05±0,02^{aA}	0,03±0,01^{aA}	0,06±0,02^{aA}
	14	0,05±0,02^{aA}	0,05±0,02^{aA}	0,03±0,01^{aA}	0,06±0,02^{aA}
Lignoceric acid, 24:0	1	0,05±0,02^{aA}	0,04±0,02^{aA}	0,03±0,01^{aA}	≤0,01
	14	0,05±0,02^{aA}	0,04±0,02^{aA}	0,03±0,01^{aA}	≤0,01
Monounsaturated fatty acids					
Myristoleic acid, 14:1	1	0,85±0,06^{aA}	1,84±0,12^{bA}	1,09±0,07^{dA}	0,55±0,04^{cA}
	14	0,80±0,06^{aA}	1,75±0,12^{bA}	1,0±0,07^{dA}	0,50±0,04^{cA}
Palmitoleic acid 16:1	1	2,49±0,19^{aA}	2,30±0,19^{aA}	3,64±0,29^{bA}	3,67±0,27^{bA}
	14	2,96±0,19^{aB}	2,15±0,19^{bA}	3,54±0,29^{cA}	3,58±0,27^{cA}
Oleic acid, 18:1	1	21,49±1,67^{aA}	20,74±1,67^{aA}	16,41±1,57^{bA}	19,67±1,71^{aA}
	14	19,49±1,67^{aA}	20,12±1,67^{aA}	16,30±1,57^{aA}	19,45±1,71^{aA}
Erucid acid, 22:1	1	≤0,01	≤0,01	≤0,01	≤0,01
	14	≤0,01	≤0,01	≤0,01	≤0,01
Polyunsaturated fatty acids					
Linoleic acid, 18:2	1	2,48±0,18^{aA}	3,57±0,31^{bA}	1,90±0,28^{aA}	2,44±0,31^{aA}
	14	1,86±0,18^{aB}	3,38±0,31^{bA}	1,75±0,28^{aA}	2,35±0,31^{cA}
Linolenic acid, 18:3	1	0,28±0,06^{aA}	4,44±0,39^{bA}	0,25±0,05^{aA}	0,37±0,05^{aA}
	14	0,26±0,06^{aA}	4,25±0,39^{bA}	0,20±0,05^{aA}	0,30±0,05^{cA}
Eicosadienoic acid, 20:4	1	0,14±0,01^{aA}	0,23±0,05^{bA}	0,11±0,04^{aA}	0,18±0,03^{bA}
	14	0,14±0,01^{aA}	0,20±0,05^{bA}	0,16±0,03^{aA}	0,11±0,04^{aA}
Eicosapentaenoic acid, 20:5	1	n.d	n.d	1,72±0,25^{bA}	2,36±0,28^{aA}
	14	n.d	n.d	1,70±0,25^{bA}	2,30±0,28^{aA}
Docosahexaenoic acid, 22:6	1	n.d	n.d	10,16±1,26^{bA}	2,41±0,23^{aA}
	14	n.d	n.d	10,15±1,26^{bA}	2,35±0,23^{aA}

Fatty acids, %	Storage day	Control sample	Yoghurt with chia oil	Yogurt with algae oil	Yoghurt with cod liver oil

n.d- not detected; a–d letters point out differences (P < 0.05) between yogurt samples; A–B letters point out differences (P < 0.05) between storage days
